# The effects of extruded corn flour on rheological properties of wheat‐based composite dough and the bread quality

**DOI:** 10.1002/fsn3.1153

**Published:** 2019-08-01

**Authors:** Huaxing Sun, Qian Ju, Jie Ma, Jincheng Chen, Yaoxi Li, Yanqiu Yuan, Yayun Hu, Kaori Fujita, Guangzhong Luan

**Affiliations:** ^1^ College of Food Science and Engineering Northwest A&F University Yangling China; ^2^ Japan International Research Center for Agricultural Science Ibaraki Japan

**Keywords:** bread, extrusion, farinographic properties, maize

## Abstract

The effects of extruded corn flour (ECF) on the rheological properties of the wheat‐based composite dough and quality of the bread were investigated. The RVA results of the composite flour with ECF showed weak thermal viscosity and resistance to starch retrogradation. Mixolab tests revealed that the water absorption capacity increased with the increasing amount of ECF, while dough development time (DT) and dough stability (ST) showed a downward trend, and the composite dough became more resistant to retrogradation. The microstructure of the composite dough showed that the presence of both ECF and unextruded corn flour (UECF) resulted in a more broken gluten matrix. The breads made from the composite flour with ECF had significantly softer texture, lower hardening percentage with storage time, darker crust color, larger specific volume, and higher sensory scores than the UECF ones. It is concluded that the extrusion of corn flour is an effective way to improve the quality of the composite bread and retard staling during storage.

## 1 INTRODUCTION

Maize (*Zea mays* L.), commonly called corn, has been ranked the first cereal crops in the world with yearly production over billion tons since 2016 (FAO, 2018). It is not only an essential cereal for the security of food and feed supply, but also the most important material for starch and related industries (Gao et al., 2018). Maize contains approximately 62% starch, 8.7% protein, and 4% fat (Hassan, Hoersten, & Ahmed, 2019) and is rich in dietary fiber, vitamins, minerals, and functional elements (phenolics, flavonoids, anthocyanins, β‐carotene, and lutein) (Zhang et al., 2016; Žilić, 2012). With the increasing awareness of health, there recently became a trend of “staplization” of minor grains, potato, or maize by introducing them into the system of staple foods dominated by wheat and rice. Such attempts were mostly focused on gluten‐free bread from minor grain, potato, and maize, or composite bread mixed with wheat (Brites, Trigo, Santos, Collar, & Rosell, 2010; Liu et al., 2017). However, the dilution and disruption of gluten network in a composite dough could decrease consistency, cohesiveness, and extensibility of the dough, and result in quality deterioration of bread on texture, taste, shelf life, appearance, and specific volume (Jafari, Koocheki, & Milani, 2018a; Moore, Juga, Schober, & Arendt, 2007). Gluten (Marchetti, Cardós, Campaña, & Ferrero, 2012), modified starches (Yousif, Gadallah, & Sorour, 2012), hydrocolloids (Hager & Arendt, 2013), and enzymes (Schoenlechner, Szatmari, Bagdi, & Tömösközi, 2013) have been used to improve the viscoelasticity of the dough and quality of bread.

Thermal treatment of material is another way to increase the dough cohesion by gelatinization of starch. Extrusion is a crucial method of hydrothermal treatment. The material undergoes the joint action of temperature, humidity, pressure, and mechanical shear during the extrusion process, which causes the starch to gelatinize and damage, the dietary fiber to dissolve, and the protein to aggregate, thus improves the consistency and cohesiveness of the dough, and modifies the functionality of cereal flour (Gómez, Jiménez, Ruiz, & Oliete, 2011; Patil, Rudra, Varghese, & Kaur, 2016; Sun et al., 2018). It has been demonstrated that extruded wheat bran and wheat germ improved dough stability and bread qualities (Gómez, González, & Oliete, 2012; Gómez et al., 2011). Ma et al. (2018) found that the dough with extruded black rice showed more viscous behavior, higher resistance, and extensibility, and the bread with extruded black rice had higher specific volume, lower bake loss, and firmness. Extrusion cooking could also improve the flavor and the overall acceptability of extruded sorghum–wheat composite bread (Jafari, Koocheki, & Milani, 2018b). The effects of extruded wheat flours (Martínez, Oliete, & Gómez, 2013), extruded finger millet (Patil et al., 2016), and extruded sorghum flour (Jafari, Koocheki, & Milani, 2017; Jafari et al., 2018a) on composite dough rheology and textural and organoleptic properties of breads also had been studied. However, the influences of extruded corn flour on the dough rheology and bread quality have not been fully understood, and more detailed information is needed to clarify the scientific facts and support its potential application.

## 2 MATERIALS AND METHODS

### 2.1 Materials

Commercial wheat flour with high gluten content (WF, 11.6% moisture, 0.45% ash, 14.6% protein, 0.96% fat, and 71.76% starch) and corn grits (12.9% moisture, 0.22% ash, 7.62% protein, 0.20% fat, and 58.5% starch) were purchased from local market.

Extrusion of corn grit was conducted with a laboratory‐scale single‐screw extruder (YJP100, Shandong University of Technology). The moisture was adjusted to reach final moisture content of 21% prior to extrusion. The extruded temperature and the screw speed were set at 100°C and 180 rpm, respectively. The circular matrix with 8 mm × 3 diameter was used. Both extruded and unextruded corn grits were ground with a universal grinder (FW‐400A, Beijing Zhongxing Weiye Instrument Co., Ltd.) for 2 min and screened through a sieve (80 mesh) to make extruded corn flour (ECF) and unextruded corn flour (UECF).

The premixed flour of WF and ECF or UECF with substitution of 10%, 20%, 30%, and 40% was thoroughly blended by rotating and shaking in a resealable plastic bag.

### 2.2 Methods

#### 2.2.1 Pasting properties of flour

Pasting properties of WF, ECF, UECF, and mixed flour were determined according to the method of LS/T 6101‐2002 (Yan, Zhang, & He, 2001) with a Rapid Viscosity Analyzer (RVA‐4500, Perten Instruments). Flour samples were weighed 3.5 g on the basis of 14% moisture and then dispersed in 25 ml of distilled water for the RVA measurement. Suspensions were held at 50°C for 1 min and then raised to 95°C at a rate of 12°C/min, held at 95°C for 2.5 min and cooled to 50°C at the same rate, and finally held at 50°C for 2 min. The stirring rate was 960 rpm for the first 10 s followed by 160 rpm for the remaining test. Peak time (PT1), pasting temperature (PT2), peak viscosity (PV), final viscosity (FV), breakdown (BD), and setback (SB) were obtained from the pasting curve.

#### 2.2.2 Mixolab measurements

A Mixolab 2 Mixing Tester (Chopin, Tripetteet Renaud) was used to study the agitation and batter characteristics of WF, ECF, UECF, and mixed flour using chopin + protocol according to ICC No. 173 (ICC Standard Methods, 2008). The following parameters were derived from the experimental curves (Rosell, Collar, & Haros, 2007): Water absorption (%) (WA) is the amount of water required to produce a torque of 1.1 ± 0.05 Nm for the dough; development time (min) (DT) is the time to reach the maximum torque at 30°C; stability (min) (ST) is the time that the torque produced could be kept at 1.1 Nm; minimum torque (Nm) (C2) is the torque generated by dough submitted to mechanical and thermal constraints; peak torque (Nm) (C3) is referred to the maximum torque produced during the heating stage; and setback (Nm) (C5‐C4) is the difference between the torque produced after cooling to 50°C (C5) and the torque after the heating period (C4).

#### 2.2.3 Microstructure of dough

The morphology of the dough was observed using a scanning electron microscope (Nova NanoSEM‐450, EI) according to the method of Ma et al. (2018). Small portions of sample were cut with a razor blade and immersed in 4% glutaraldehyde at 4°C for 12 hr or more; then dipped four times with phosphate buffer pH 6.8 at a concentration of 0.1 mol/L for 10 min; after rinsing, embedded in a graded ethanol series (30%, 50%, 70%, and 90%) for 15 min at each gradation; and then embedded in 100% ethanol for 30 min three times. Following that, the samples were dried by supercritical CO_2_ and then coated with gold particles for 4 min. The micrographs were taken at 1,000 magnification.

#### 2.2.4 Preparation of dough and bread

A straight dough method (10‐10B, AACC International, 2000) with minor modification was used for preparation of bread. The dough was made using the following formula: WF or premixed flours (300 g), sugar (30 g), salt (2.4 g), instant dried yeasts (5.4 g), lard (9 g), and skimmed milk powder (12 g). The amount of water was calculated from water absorption obtained by Mixolab measurements. All materials except lard were added in a five‐speed dough mixer (SM‐1688, Shepherd Wang Electrical Hardware Co., Ltd.) and mixed following a schemed sequence as follows: speed of level 2 for 2 min, level 3 for 1 min, level 2 for 1 min after adding lard, level 3 for 1 min, and level 4 for 1 min. The dough was fermented at 30°C and relative humidity of 85% (RH) for 90 min. During the fermentation, the punching was done at 55 min. The dough was divided into two pieces and rolled at the distance of 7.5 and 5.5 mm using a electric roller (300/100 type), respectively. The sheets of dough were rolled up by hand and placed in stainless baking molds (15 × 7 cm^2^ in top, 15 × 6 cm^2^ in bottom, and 6.5 cm in depth). After proofing for 40 min at 30°C and RH of 85%, the dough was baked in an oven for 20 min (upper temperature 190°C and bottom temperature 200°C). The breads were removed from molds and cooled for 2 hr at 20°C before further analyses. The specific volume of bread was determined by the rapeseed displacement method and calculated in ml/g.

#### 2.2.5 Hardness of bread

Textural properties of the breads were evaluated using Texture Analyzer (TA‐XA Plus, Stable Micro Systems, Ltd.) according to the method of 74‐09 (AACC, 2000). The bread was sliced mechanically for 12.5 mm thick from the central loaf. Two slices were stacked together for test sample. A 36‐mm‐diameter cylindrical aluminum probe was used in a double compression test with 40% penetration depth. The test speed was 1mm/s and with a 5‐s delay between first and second compressions. The bread was stored at 20°C in plastic bags for 0, 1, 3, 5, and 7 days, respectively, to measure the change of hardness.

#### 2.2.6 Color measurement

The color of bread was measured using colorimeter (Ci7x00; X · rite, Glanville) with measurement area ø = 10 mm and Illuminant D65. The color values of the central point on each quarter of crust or crumb were measured and recorded according to color space of CIE L*a* b*. Every measurement was duplicated for seven times.

#### 2.2.7 Sensory evaluation

Sensory evaluation of breads was performed with a group of twenty trained panelists using nine‐point hedonic scale according to the method of Patil et al. (2016). The bread samples were coded with random three‐digit numbers. The panelists were introduced to rinse and swallow water between samples. The following sensory indicators were evaluated: appearance, flavor, texture, taste, and overall acceptability. The score ranged from 1 (dislike extremely) to 9 (like extremely).

#### 2.2.8 Statistical analyses

Measurements were performed at least in duplicate and analyzed using analysis of variance (ANOVA); then, the results were expressed as mean value ± standard deviation. A Tukey's multiple range test was conducted to establish the significant differences among experimental mean values (*p* < .05). All statistical analyses were performed using DPS version 7.05 for windows.

## 3 RESULTS AND DISCUSSION

### 3.1 Pasting properties of flour

The pasting parameters of WF, ECF, UECF, and composite flours are shown in Table 1. The pasting properties could be used for evaluating the edible quality of cereals (Shi et al., 2016). ECF had the lowest PT1, PT2, FV, and SB, while UECF had the highest PV, FV, and SB. Compared with WF and substituted UECF, the composite flour with ECF had higher PT2, but lower PT1, PV, FV, BV, and SB. With increasing level of ECF substitution, there was a significant (*p* < .05) decrease in PV, FV, BD, and SB, indicating a severe breakage of the amylose chain, a loss of retrograde ability (Martínez, Rosell, & Gómez, 2014), and a potential ability of antistaling. This may be attributed to molecular degradation (Zeng, Gao, Li, & Liang, 2011) and pregelatinization of starch (Martínez et al., 2013) during extrusion. The substitution of UECF resulted in a slight increase in PV, FV, and SB, showing higher viscous behavior. Similar results were obtained by Brites et al. (2010) and Martínez and El‐Dahs (1993).

**Table 1:** Pasting properties of WF, ECF, UECF and composite flour of WF and ECF or UECF

Sample	PT1 (min)	PT2 (°C)	PV (cp)	FV (cp)	BD (cp)	SB (cp)
ECF	1.10 ± 0.04e	50.63 ± 0.74h	545.00 ± 36.77e	308.50 ± 12.02j	368.50 ± 36.06e	132.00 ± 12.73g
UECF	5.27 ± 0.00d	77.03 ± 0.67g	1794.00 ± 11.31a	2,895.00 ± 50.91a	375.00 ± 5.66e	1,476.00 ± 45.25a
WF	5.90 ± 0.04a	87.25 ± 0.07c	1622.50 ± 17.68b	2020.50 ± 19.09e	655.50 ± 0.71a	1,053.50 ± 2.12b
10% ECF	5.63 ± 0.14bc	87.63 ± 0.60bc	1,194.00 ± 16.97c	1585.50 ± 31.82f	459.00 ± 2.83d	850.50 ± 12.02c
20% ECF	5.50 ± 0.04c	89.28 ± 0.60ab	837.50 ± 0.71d	1,167.00 ± 8.49g	286.50 ± 14.85f	616.00 ± 22.63d
30% ECF	5.24 ± 0.05d	89.60 ± 0.00a	571.00 ± 12.73e	844.00 ± 21.21h	193.00 ± 7.07g	466.00 ± 15.56e
40% ECF	5.07 ± 0.00d	90.10 ± 0.57a	396.50 ± 0.71f	604.50 ± 2.12i	126.50 ± 2.12h	334.50 ± 0.71f
10% UECF	5.77 ± 0.05ab	86.40 ± 0.00cd	1623.00 ± 29.70b	2056.50 ± 33.23de	633.00 ± 9.90a	1,066.50 ± 13.44b
20% UECF	5.67 ± 0.00bc	84.68 ± 0.04de	1635.50 ± 7.78b	2,145.50 ± 2.12cd	610.50 ± 6.36ab	1,120.50 ± 0.71b
30% UECF	5.67 ± 0.00bc	84.40 ± 0.57e	1642.50 ± 13.44b	2,236.50 ± 23.33bc	567.00 ± 1.41bc	1,161.00 ± 11.31b
40% UECF	5.60 ± 0.00bc	82.28 ± 0.04f	1658.50 ± 13.44b	2,309.00 ± 67.88b	521.00 ± 1.41c	1,171.50 ± 82.73b

Note

Data are shown as the mean ± standard deviation. Values in the same column with different letters are significantly different at *p* < .05. 10%, 20%, 30% and 40%, levels of substitution by ECF or UECF.

Abbreviations: BD, breakdown; ECF, extruded corn flour; FV, final viscosity; PT1, peak time; PT2, pasting temperature; PV, peak viscosity; SB, setback; UECF, unextruded corn flour; WF, wheat flour.

### 3.2 Mixolab dough properties

The parameters WA, DT, ST, C2, C3, and C5‐C4 are shown in Table 2. The dough from composite flour with ECF had higher WA than UECF and wheat dough. This may be due to the structure disruption and the swelling of highly degraded starch (Zeng et al., 2011). Higher amount of water for dough forming is beneficial to improve the bread yield. With regard to DT, the substitution of corn flour decreased the time to reach the maximum consistency of dough (except for 10% ECF). In contrast with our results, Jafari et al. (2017) found that the development time increased with addition of extruded sorghum. Martínez et al. (2013) observed that there were no significant differences in dough development time with the addition of extruded wheat flours. This may be caused by the different nature of cereal materials. Khoshgozaran‐Abras, Azizi, Bagheripoor‐Fallah, and Khodamoradi (2014) found that the lower DT was more suitable to get appropriate dough. Therefore, dough made with substituted ECF and UECF was desired. ST was observed to decrease with the addition of ECF. Dough stability relies on the characteristics of the protein network (Konopka, Fornal, Abramczyk, Rothkaehl, & Rotkiewicz, 2004). High stability values are usually associated with flour strength (Marco & Rosell, 2008). The addition of ECF and high dose of UECF decreased the dough stability, which may be attributed to the dilution of gluten along with the addition of ECF and UECF, thus reducing the cohesion of dough. The addition of ECF decreased the value of C2, while a decrease was found for UECF. C2 value refers to the protein weakening caused by mechanical and thermal limitations. Higher C2 torque showed the lower protein weakening (Moza & Gujral, 2018). The point of C3 is related to starch gelatinization. The difference between the C5 and C4 value indicates degree of starch retrogradation (Torbica, Hadnađev, & Dapčević, 2010). The values of torque at C3 point and the values of C5‐C4 decreased with the increasing amount of ECF in composite dough, indicating that it was more stable and difficult to retrograde. This is consistent with the above result of RVA test.

**Table 2:** The Mixolab thermomechanical properties of WF, ECF and composite dough of WF and ECF or UECF

Samples	WA	DT (min)	ST (min)	C2 (*N*·m)	C3 (*N*·m)	C5‐C4 (*N*·m)
ECF	110.0	7.86 ± 0.46a	8.16 ± 0.04b	0.28 ± 0.01e	0.33 ± 0.01i	0.32 ± 0.01e
WF	61.9	5.07 ± 3.25ab	10.25 ± 0.21a	0.42 ± 0.01c	1.62 ± 0.01d	0.85 ± 0.05ab
10% ECF	62.3	5.88 ± 0.78a	9.35 ± 0.25ab	0.32 ± 0.01d	1.45 ± 0.00e	0.53 ± 0.00d
20% ECF	68.0	0.53 ± 0.01c	0.42 ± 0.05d	0.15 ± 0.00f	1.21 ± 0.00f	0.24 ± 0.00f
30% ECF	71.0	0.84 ± 0.01bc	0.82 ± 0.00cd	0.14 ± 0.00f	0.85 ± 0.01g	0.22 ± 0.00f
40% ECF	78.8	0.60 ± 0.04c	0.48 ± 0.08d	0.08 ± 0.00g	0.55 ± 0.11h	0.09 ± 0.00g
10% UECF	60.0	1.59 ± 0.01bc	9.90 ± 0.07ab	0.48 ± 0.01b	1.78 ± 0.05c	0.91 ± 0.02a
20% UECF	59.5	1.62 ± 0.12bc	10.04 ± 0.62a	0.50 ± 0.00ab	1.83 ± 0.01bc	0.81 ± 0.00b
30% UECF	59.0	1.40 ± 0.11bc	8.94 ± 1.22ab	0.53 ± 0.01a	1.96 ± 0.01ab	0.72 ± 0.00c
40% UECF	58.5	1.14 ± 0.12bc	2.33 ± 0.04c	0.51 ± 0.01ab	2.02 ± 0.00a	0.65 ± 0.00c
UECF	–	–	–	–	–	–

Note

Data are shown as the mean ± standard deviation. Values in the same column with different letters are significantly different at *p* < .05. –, values not detectable; 10%, 20%, 30% and 40%, levels of substitution by ECF or UECF; C2, minimum torque during temperature increase; C3, maximum torque during 90°C stage; C5‐C4, the difference between the torque produced after cooling to 50°C (C5) and the torque after the heating period (C4).

Abbreviations: DT, development time; ECF: extruded corn flour; UECF: unextruded corn flour; ST, stability; WA, water absorption; WF: wheat flour.

### 3.3 Scanning electron microscopy (*SEM*) of composite dough

**Figure 1:**
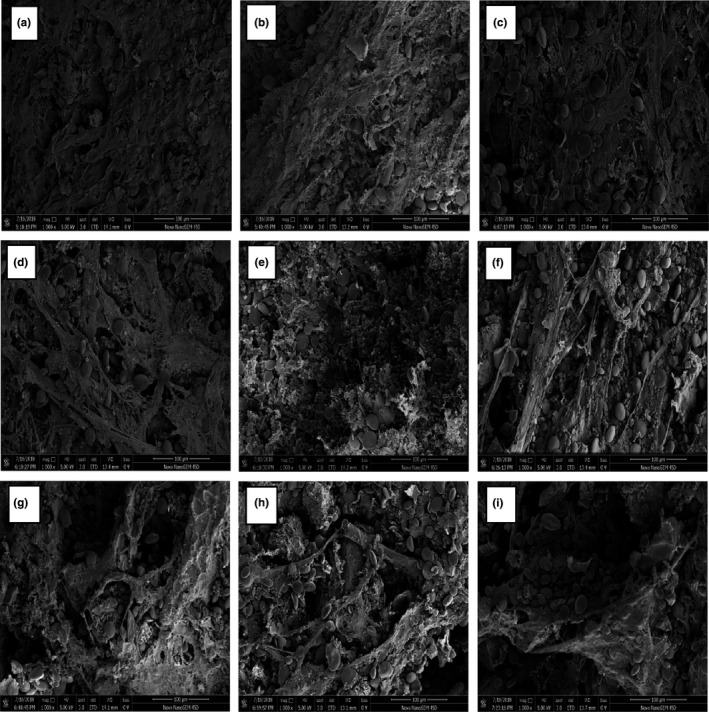
The morphology of WF dough and composite dough of WF and ECF or UECF. (a) wheat dough at 1,000×; (b–e) 10%, 20%, 30%, and 40% of ECF‐wheat composite dough at 1,000×; (f–i) 10%, 20%, 30%, and 40% of UECF‐wheat composite dough at 1,000×. ECF, extruded corn flour; UECF, unextruded corn flour

The morphology of composite dough observed by *SEM* is presented in Figure 1. The wheat dough (a) had a compact network structure with starch granules immersed inside. While with the increasing level of ECF and UECF substitution, the dough matrix became discontinuous and incompact. The composite dough with UECF (f–i) had more complete starch granules than the ECF ones (b–e). This could be attributed to the destruction of the starch structure during extrusion (Zhang et al., 2016). Moreover, the damaged starch granules would be helpful to enzymatic hydrolysis. Martínez et al. (2013) also observed that the dough mixed with extruded wheat flour formed less compact network structure. The presence of ECF and UECF resulted in a more broken gluten matrix, similar to the report by Feng and Sun (2013). The dough with UECF substitution had larger gaps and worse structure; this may be due to the dilution of gluten protein caused by UECF. However, the dough with ECF substitution had relatively better structure than the UECF ones, which had little gaps and more sticky substances, and this might because the starch was gelatinized during extrusion and acted as hydrocolloid that could improve the dough structure. These results indicated that the microstructure of composite dough with ECF substitution could be greatly improved.

### 3.4 Hardness of bread

The effects of substitution of corn flour on the hardness of crumb are shown in Table 3. With respect to the fresh bread, both the addition of ECF and UECF increased the hardness of bread. The hardness of composite bread with unextruded corn flour (UECB) was significantly higher than wheat bread (WB) and composite bread with extruded corn flour (ECB). Similar results were found for extruded flour of maize (Ozola, Straumite, & Klava, 2011), wheat (Martínez et al., 2013), bran (Gómez et al., 2011), and finger millet (Patil et al., 2016). The softer crumb of ECB may be due to the high water retention capacity, gelatinization, and damaged starch (Patil et al., 2016). The hardness of all samples increased significantly during storage, although at a different degree. The composite breads with 20% and 30% ECF addition showed the lowest hardening percentage (data not shown), indicating that the extruded corn flour could slow the staling rate of bread. This may be due to the fact that ECF was easy to gelatinize and had the ability to retard aging according to the results of RVA and Mixolab. Moreover, the higher water retention capacity of ECF and the higher water content of ECB may explain the retrogradation ability of ECB. Tsai et al. (2012) also found that the texture property of wheat flour bread was improved with the substitution of 15% rice porridge during storage, due to the gelatinization of rice starch granules.

**Table 3:** Hardness changes of WB, ECB, and UECB during storage

Texture property	Bread samples	Days of storage				
		0	1	3	5	7
Hardness	WB	403.52 ± 7.57d	807.00 ± 38.18fg	1,121.50 ± 108.18ef	1,324.00 ± 93.33e	1554.00 ± 35.35de
	10% ECB	472.46 ± 2.65cd	991.81 ± 15.95de	1,433.09 ± 50.52de	1,770.94 ± 15.44cd	1962.58 ± 50.10d
	20% ECB	476.50 ± 0.70cd	705.50 ± 16.26g	1,087.42 ± 19.00f	1,251.65 ± 179.63e	1,273.70 ± 42.07e
	30% ECB	547.50 ± 9.38c	890.01 ± 32.45ef	1,003.53 ± 96.81f	1,138.77 ± 57.08e	1,237.66 ± 31.12e
	40% ECB	694.00 ± 49.49b	1,229.41 ± 37.59c	1592.50 ± 60.10cd	2020.65 ± 39.87c	2,660.00 ± 35.15c
	10% UECB	522.285 ± 9.30c	1,095.222 ± 547.39cd	1,315.23 ± 9.81def	1645.28 ± 1.08d	1815.60 ± 3.54d
	20% UECB	513.50 ± 41.71c	1,080.5 ± 3.53cd	1887.00 ± 15.55c	2,911.50 ± 4.95b	3,598.50 ± 77.07b
	30% UECB	1,213.73 ± 8.52a	1959.4 ± 89.62b	2,851.10 ± 10.48b	3,019.64 ± 78.15b	3,372.20 ± 10.08b
	40% UECB	1,278.59 ± 32.53a	2,618.00 ± 31.75a	3,952.10 ± 184.33a	3,983.90 ± 80.80a	4,876.16 ± 316.37a

Note

Data are shown as the mean ± standard deviation. Values in the same column with different letters are significantly different at *p* < .05. 10%, 20%, 30% and 40%, levels of substitution by ECB or UECB.

Abbreviations: ECB, composite bread with extruded corn flour; UECB, composite bread with unextruded corn flour; WB, wheat bread.

### 3.5 Specific volume and color measurement

**Figure 2:**
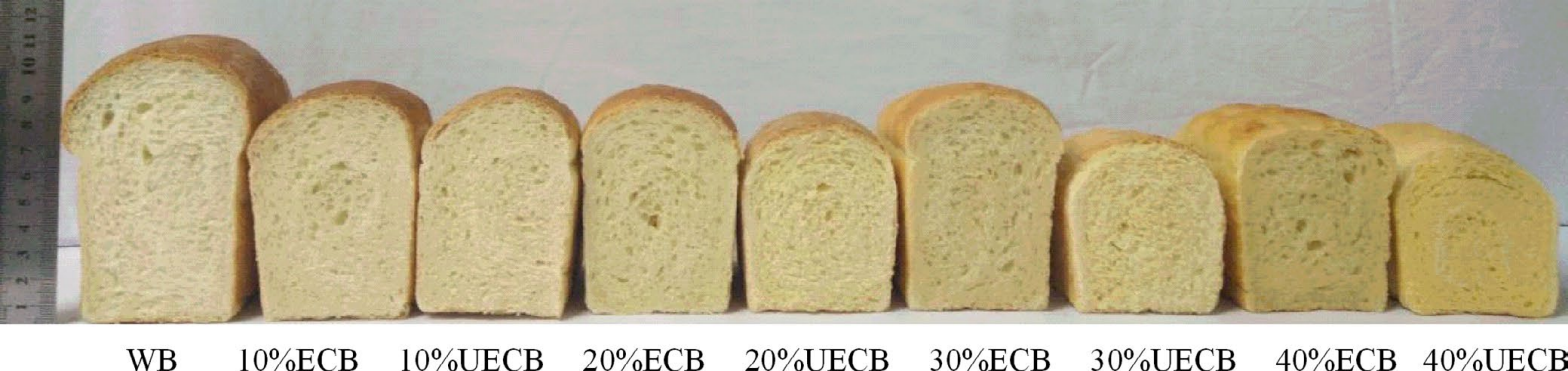
Images of WB, ECB, and UECB. 10%, 20%, 30%, and 40%, levels of substitution by ECB or UECB; ECB, composite bread with extruded corn flour; UECB, composite bread with unextruded corn flour; WB, wheat bread

The effects of ECF and UECF on the specific volume and bread color are presented in Table 4 and Figure 2. With the addition of ECF and UECF, the specific volume of bread decreased. The specific volume of UECB was lower than ECF at all levels of substitution. Patil et al. (2016) and Martínez et al. (2013) also observed that the higher proportion of damaged starch in extruded finger millet flour and wheat flour was beneficial for better yeast activity and production of fermentable sugars, and the increased gas production and retention made ECB have higher specific volume. The partial gelatinization of starch could increase the consistency of dough and may capture the gas during mixing and baking (Bourekoua, Benatallah, Zidoune, & Rosell, 2016), which thus also improved the specific volume and quality of ECB.

**Table 4:** Specific volume and color parameters of WB, ECB, and UECB

Samples	Specific volume	Color value of bread crust			Color value of bread crumb		
		L*	a*	b*	L*	a*	b*
WB	3.56 ± 0.05a	53.75 ± 0.69de	12.69 ± 0.13b	17.99 ± 0.48e	70.23 ± 1.45a	−0.82 ± 0.02f	7.96 ± 0.04g
10% ECB	2.66 ± 0.02b	52.03 ± 0.50f	11.20 ± 0.24c	15.55 ± 0.35f	68.91 ± 0.59ab	−0.75 ± 0.11f	10.47 ± 0.18f
20% ECB	2.56 ± 0.05b	53.01 ± 0.18ef	12.38 ± 0.16b	18.25 ± 0.31e	64.75 ± 1.56bcd	−0.19 ± 0.03d	10.79 ± 0.54ef
30% ECB	2.41 ± 0.01c	54.65 ± 0.59d	13.28 ± 0.30a	21.46 ± 0.22cd	63.05 ± 0.28d	0.22 ± 0.092c	11.68 ± 0.30e
40% ECB	1.68 ± 0.03f	59.89 ± 0.06b	8.99 ± 0.35e	23.59 ± 0.15b	62.68 ± 1.86d	0.27 ± 0.03c	14.21 ± 0.20cd
10% UECB	2.60 ± 0.03b	58.45 ± 0.43c	12.09 ± 0.10b	20.75 ± 0.22d	67.60 ± 0.06abc	−0.38 ± 0.03e	13.51 ± 0.17d
20% UECB	2.22 ± 0.01d	59.57 ± 0.12bc	11.35 ± 0.57c	21.91 ± 0.74c	65.97 ± 0.42bcd	0.22 ± 0.06c	14.72 ± 0.22c
30% UECB	2.04 ± 0.01e	63.66 ± 0.76a	10.83 ± 0.32c	23.67 ± 0.31b	64.29 ± 0.60cd	1.17 ± 0.03b	17.45 ± 0.47b
40% UECB	1.67 ± 0.02f	64.47 ± 0.27a	10.04 ± 0.10d	25.79 ± 0.25a	63.07 ± 0.81d	1.64 ± 0.07a	19.60 ± 0.54a

Note

Data are shown as the mean ± standard deviation. Values in the same column with different letters are significantly different at *p* < .05. 10%, 20%, 30% and 40%, levels of substitution by ECB or UECB.

Abbreviations: ECB, composite bread with extruded corn flour; UECB, composite bread with unextruded corn flour; WB, wheat bread.

With increase in substitutive level, the value L* and b* of both ECB and UECB crust increased, respectively, while the value a* of UECB crust decreased. Both the values L* and b* of ECB crust were lower than those of UECB at all levels of substitution. The value L* of WB crust was higher than that of 10% ECB, but lower than those of the 40% ECB and all samples of the UECB group, and had no difference to the 20% ECB and 30% ECB. This was mainly due to the higher loaf height (Figure 2) narrowed its distance to the upper heating element. Another reason might be attributed to the sufficient reducing sugars in ECB for Maillard reaction (Martínez et al., 2013). The lower L* value of ECB crust than UECB crust indicated that ECB had darker crust color. The difference of value a* between crust of WB and the ECB or UECB was not so big. The value b* of WB crust was lower than all samples of ECB or UECB except 10% ECB, which reflected the nature yellow color of corn.

With increase in substitutive level, the values a* and b* of both ECB and UECB crumb increased, respectively, while the value L* decreased. The change in value a* was slight, especially for the ECB group. Both the values a* and b* of ECB crumb were lower than that of UECB crumb at all levels of substitution, while the L* values were all higher. The difference of both value L* and a* between crumb of ECB and UECB at all levels of substitution was slight. The main change in color of crumb was in yellowness. The color of crumb is mainly determined by materials (Turkut, Cakmak, Kumcuoglu, & Tavman, 2016). The crumbs of both ECB and UECB were in yellow color, which reflected the nature color of corn.

### 3.6 Sensory evaluation of bread

**Figure 3:**
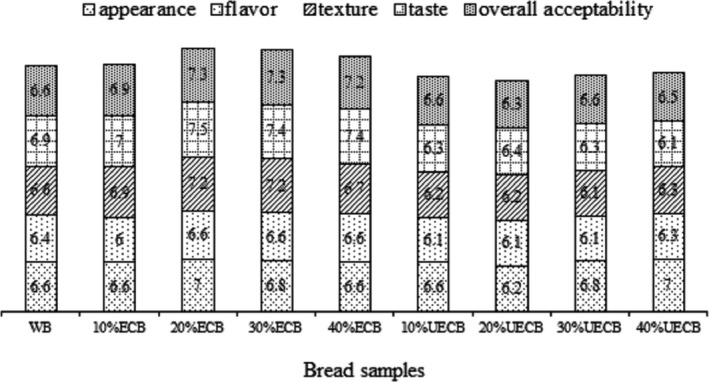
Sensory evaluation of WB, ECB, and UECB. 10%, 20%, 30%, and 40%, levels of substitution by ECB or UECB; ECB, composite bread with extruded corn flour; UECB, composite bread with unextruded corn flour; WB, wheat bread

The sensory scores of composite breads were shown in stacked column graph (Figure 3). Texture and taste scores of ECB were higher than that of UECB, which accounted for the overall high acceptability score of the extruded corn bread. The addition of ECF and UECF had little effect on the appearance of the bread. It can be observed that higher substitution than 10% of ECF led to a slight increase in flavor score. The bread crumb of ECB was softer than WB and UECB, which had the higher score than UECB. Soft bread crumb usually got high score in sensory evaluation (Torbica et al., 2010). Taste scores of ECB were also higher than UECB, which could be attributed to slight sweetish taste of extruded flour (Patil et al., 2016). All the samples were considered acceptable with the overall scores higher than 5, while the ECB got the highest. This result could be contributed to the opinion that hydrothermal process and extrusion treatment could have a positive impact on the quality of composite bread (Patil et al., 2016; Stokić et al., 2015).

## 4 CONCLUSION

The composite flour with ECF showed higher pasting temperature; but lower values of peak time, peak viscosity, final viscosity, and breakdown from the results of RVA tests; higher water absorption capacity; shorter dough development time; and lower dough stability from the results of Mixolab tests. The microstructure morphology of the composite dough structure showed that the presence of both ECF and UECF resulted in a more broken gluten matrix, and more entire starch granules were retained for the UECF one. The above facts indicated that the composite flour with ECF would result in softer dough with lower proofing resistance but weak ability of gas retaining during fermentation. The interactive balance of the above factors may be the reason why ECB had larger specific volume than the UECB ones but lower than WB.

Both the results from the RVA and Mixolab tests showed the resistance to starch retrogradation of ECF. The ECB with 20%, 30%, and 40% addition had significantly softer texture than that of UECB, and the breads with 20% and 30% substitution of ECF were significantly softer than both WB and UECB during storage. The above facts sufficiently approved the antistaling ability of ECF.

The bread from composite flour with ECF had lower hardening percentage with storage time, darker crust color, larger specific volume, and higher sensory scores than the UECB. Consequently, it can be concluded that the extrusion of corn flour is an effective way to improve the quality of composite bread.

## CONFLICT OF INTEREST

The authors declare no conflict of interest.

## ETHICAL APPROVAL

This study has nothing to do with human and animal testing.
